# The Impact of Time of Day on Energy Expenditure: Implications for Long-Term Energy Balance

**DOI:** 10.3390/nu11102383

**Published:** 2019-10-06

**Authors:** Emma Shaw, Gloria K.W. Leung, Jessica Jong, Alison M. Coates, Rochelle Davis, Merran Blair, Catherine E. Huggins, Jillian Dorrian, Siobhan Banks, Nicole J. Kellow, Maxine P. Bonham

**Affiliations:** 1 Department of Nutrition, Dietetics and Food, Monash University, Melbourne, VIC 3168, Australia; emma.shaw@monash.edu (E.S.); jjon29@student.monash.edu (J.J.); rochelle.davis@monash.edu (R.D.); Merran.Blair@monash.edu (M.B.); kate.huggins@monash.edu (C.E.H.); nicole.kellow@monash.edu (N.J.K.); 2 Alliance for Research in Exercise, Nutrition and Activity (ARENA), School of Health Sciences, University of South Australia, Adelaide, SA 5001, Australia; alison.coates@unisa.edu.au; 3 Behaviour-Brain-Body Research Centre, School of Psychology, Social Work and Social Policy, University of South Australia, Adelaide, SA 5072, Australia; Jill.Dorrian@unisa.edu.au (J.D.); siobhan.banks@unisa.edu.au (S.B.)

**Keywords:** basal metabolic rate, energy expenditure, circadian rhythms, substrate oxidation, meal timing

## Abstract

There is evidence to indicate that the central biological clock (i.e., our endogenous circadian system) plays a role in physiological processes in the body that impact energy regulation and metabolism. Cross-sectional data suggest that energy consumption later in the day and during the night is associated with weight gain. These findings have led to speculation that when, as well as what, we eat may be important for maintaining energy balance. Emerging literature suggests that prioritising energy intake to earlier during the day may help with body weight maintenance. Evidence from tightly controlled acute experimental studies indicates a disparity in the body’s ability to utilise (expend) energy equally across the day and night. Energy expenditure both at rest (resting metabolic rate) and after eating (thermic effect of food) is typically more efficient earlier during the day. In this review, we discuss the key evidence for a circadian pattern in energy utilisation and balance, which depends on meal timing. Whilst there is limited evidence that simply prioritising energy intake to earlier in the day is an effective strategy for weight loss, we highlight the potential benefits of considering the role of meal timing for improving metabolic health and energy balance. This review demonstrates that to advance our understanding of the contribution of the endogenous circadian system toward energy balance, targeted studies that utilise appropriate methodologies are required that focus on meal timing and frequency.

## 1. Introduction

Many physiological processes in the human body, including energy regulation and metabolism, are governed by endogenous circadian rhythms, ensuring that they occur at the most appropriate time of the 24-h day [1,2]. These rhythms regulate the production of hunger/satiety hormones ghrelin and leptin as well as other hormones such as corticosterone and insulin [3]. Optimal physiology and metabolism are compromised by staying awake and eating during a circadian phase that is set for sleeping and fasting (i.e., nighttime for humans). Unusual feeding times may result in alterations to total energy expenditure (TEE), dysregulation of feeding behaviours, changes in appetite stimulating hormones and glucose metabolism [4,5,6,7]. 

In populations who often eat at night, such as shift workers, increased risks of obesity are observed, with higher rates of type 2 diabetes and cardiovascular disease also reported even after adjustment for confounders such as age and physical activity [8,9]. Whilst an increased energy intake is a logical explanation for the observed weight gain and incidence of chronic disease, in populations such as shift workers, this has not been supported by a recent systematic review and meta-analyses [10] which concluded that total energy intake of shift workers was similar to day working counterparts. If increases in total energy intake are not responsible for weight gain and increased risk of associated chronic illness, then perhaps the explanation may be more related to the changes in the times of day at which shift workers perform key behaviours related to energy expenditure and metabolism, including patterns of eating, activity and rest.

Indeed, food intake timing has been identified as important for weight regulation, even in non-shift working populations. In the UK British Birth Cohort an observed trend towards higher energy consumption in the latter half of the day has been reported [11] and those who prioritise energy intake to later in the 24-h day are also more likely to be overweight [12]. Changes in the timing of food intake in these studies is also associated with differences in the activity/rest pattern relative to food intake. In other words, in the general population, and especially in shift workers, we see changes in the timing of energy intake and expenditure that may have negative impacts on metabolism and health. Therefore, the aim of this review is to detail current understanding of daily rhythms of involuntary energy expenditure (EE) (resting and/or after eating) to direct future research aimed at supporting health in a 24-h society. In this review we report specifically on studies that have measured basal or resting metabolic rate (RMR) as a function of TEE across the 24-h day, in order to describe usual TEE rhythmicity in the absence of circadian disruption. 

We also examine the literature pertaining to the impact of time of day on EE after eating, also known as the thermic effect of food (TEF) or diet induced thermogenesis (DIT). Experimental studies that have induced circadian disruption through simulated night shift or desynchronisation protocols and measured EE have also been included here. In addition, this review will briefly comment on the circadian regulation of other physiological processes that may affect EE, including energy storage, digestion regulation, the microbiome and sleep. Physical activity, as a voluntary component of TEE, will not be considered.

## 2. Generation of Circadian Rhythms

**Figure 1:**
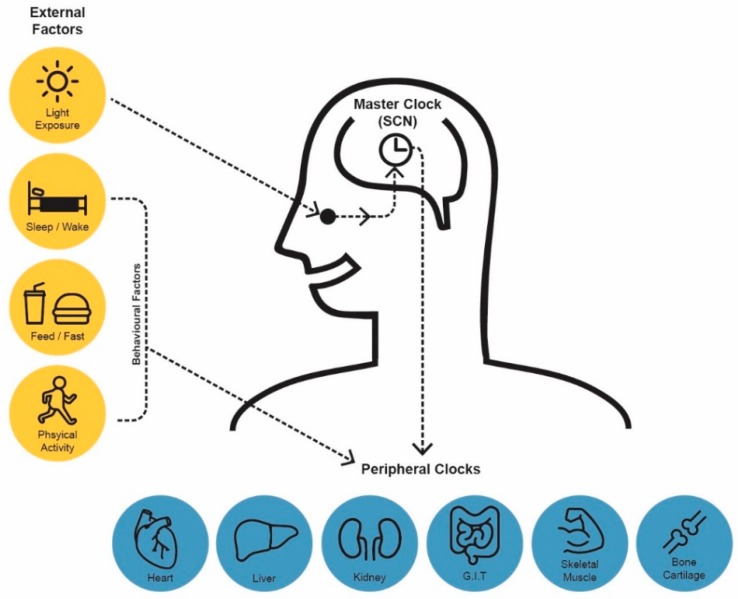
Human Circadian System: Comprised of a central clock and peripheral clocks. The central clock located in the hypothalamus is entrained by light. Peripheral clocks are influenced by physiological/behavioural factors such as sleeping and eating. Synchonisation of peripheral clocks, under the orchestration of the central clock, is essential to achieve circadian alignment.

Many homeostatic processes in humans exhibit predictable daily oscillations that approximate 24-h and are known as circadian rhythms [13]. Circadian rhythms are diurnal in that they occur approximately every 24-h, but are generated endogenously and persist in the absence of external cues such as light. Diurnal rhythms are any rhythms that present peaks and troughs and complete a cycle in approximately 24-h and are driven either by the circadian clock and/or cues from the environment, e.g., light and behavioural factors such as eating [5]. A hierarchy of circadian clocks are responsible for the generation and synchronisation of circadian rhythms to the external environment. The master circadian clock is located in the suprachiasmatic nuclei (SCN) of the hypothalamus and is entrained by light. Accordingly, the master clock synchronises behavioural and metabolic rhythms to the light/dark cycle [14,15]. In diurnal animals, i.e., humans, periods of activity and feeding are timed to occur during the daylight hours. This is initiated at a molecular level via the SCN’s coordination of peripheral circadian clocks [16] located in almost every cell throughout the body [17] (Figure 1). 

Circadian rhythms in physiological processes are regulated by oscillations in the expression of core clock genes [18]. Transcription factors CLOCK and BMAL1 heterodimerise and initiate transcription of the period (*PER 1–3*) and the cryptochrome (*CRY 1* and *2*) genes. These protein products accumulate in the cytosol and repress the action of the CLOCK–BMAL1 heterodimer upon re-entry to the nucleus [19,20]. This auto regulatory feedback loop is bolstered by secondary feedback arms in which nuclear receptors REV–ERBα and REV–ERBβ directly repress, and RORα and RORβ activate, *BMAL1* expression through competitive binding at retinoic acid-related orphan receptor binding elements in the promotor region [21]. Together, these transcriptional feedback loops represent an endogenous molecular pacemaker with one transcription loop generating a 24-h oscillation.

Circadian clocks are influential in the circadian regulation of metabolic processes, beginning at the level of transcription through clock-controlled genes [21]. Indeed, ~80% of all protein coding genes exhibit circadian rhythms in expression, albeit not in all tissues simultaneously [22]. In mammals, in addition to signals from the SCN [23], the feeding/fasting cycle has also been shown to act as an external cue, or zeitgeber, in entraining peripheral clocks [24]. This two-way relationship between metabolic regulation and peripheral clocks has been discussed extensively in a recent review by Reinke and Asher [25]. Feed/fast cycles that are inverted from the light/dark cycle actuate desynchrony of certain peripheral clocks to the master clock [26,27,28]. Desynchrony of central and peripheral clock rhythms is implicated in the aetiology of disease.

As diurnal animals, feeding and activity in humans usually occurs during the day, while fasting and resting are favoured during the night. In support of this pattern, many physiological processes have peaks and nadirs over the 24-h day. Such diurnal differences in RMR and TEF over the day/night are the focus of this review. Some work practises, such as night work, cause a circadian misalignment of eating and sleeping patterns. Studies that examine metabolic responses after disruption to usual circadian rhythms will also be discussed below. 

## 3. Components and Assessment of Total Energy Expenditure (TEE)

**Figure 2:**
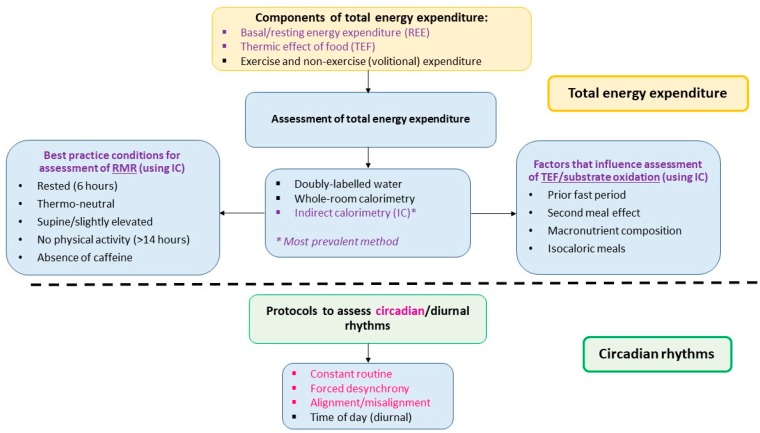
Conditions required to assess the existence of diurnal/circadian rhythms in energy expenditure. The top section of the figure presents components of total energy expenditure and the methodologies and conditions required for assessment of energy expenditure (specifically RMR). To accurately assess TEF, certain factors such as macronutrient composition of meal need to be considered. The bottom section lists study protocols used to determine circadian and diurnal rhythms. Diurnal rhythmicity can be determined using a calorimetry approach (typically IC) and time of day protocols. Circadian rhythmicity requires a forced desynchrony approach with a calorimetry technique (typically IC).

EE is quantified on the basis of heat production and can be measured using a variety of methodologies. The most precise is doubly labelled water which gives an estimate of TEE over a longer period of time [29], but individual components of TEE (see below) cannot be measured. Assessment of RMR and TEF are typically measured indirectly (via real-time measurement of oxygen consumption and carbon dioxide production) using metabolic carts or whole-body calorimetry [29] over shorter periods of time. Of the components of TEE that can be measured using these approaches RMR makes up the largest proportion; approximately two-thirds. RMR is the energy expended at rest for normal cellular functioning and therefore varies largely between individuals. Body size and composition accounts for a large portion of this variability [30], with the majority (~63%) of the between-subject variability explained by fat-free mass (FFM) and only a very small amount explained by fat mass (~6%) [30]^.^ Further, between-subject variability can also be explained independently by sex and age [31]. Commonly, RMR is measured rather than basal metabolic rate (BMR) due to the strict conditions under which BMR is measured, for example the subject must have maintained a prolonged fast (at least nine hours) and be at rest upon waking [32]. A systematic review undertaken in 2006 [33] provides useful guidance for assessment of RMR. To achieve the best predictor of a true resting state a minimum 6 h fast after eating, a room temperature between 20 °C to 25 °C, avoidance of caffeine the night before, supine or slightly elevated position and absence for vigorous activity for 14 h prior to testing are suggested conditions to meet. The energy expended during processes of digestion and absorption following a meal is largely affected by diet composition [33]. A component of TEF, substrate oxidation, refers to the oxidation of macronutrients and can be measured during indirect calorimetry by calculation of the respiratory quotient (RQ), a ratio of carbon dioxide exhaled to oxygen inhaled [34]. Carbohydrate and protein are preferentially oxidised (due to the body’s limited storage capacity) in comparison to fat which is easily stored [35]. The magnitude of the increase in TEF is therefore influenced by the composition of the meal consumed. Similar to RMR, there are factors that need to be considered when measuring the TEF, especially if the assessment is not after waking (see Figure 2). 

The third factor that contributes to energy expended is physical activity with energy lost during both volitional activity, such as physical exercise, and non-volitional activities, such as maintaining posture. Activity thermogenesis (which includes volitional and non-volitional activities) can account for between 20%–30% of TEE, however, is highly dependent on intensity and duration of the activity [36] and not a focus of this review. As collectively RMR and TEF contribute approximately 80% to TEE, any alteration to these two components of TEE do have the potential to impact energy balance, although within the context of this review much of the evidence to date has focused on the TEF.

## 4. Diurnal Variations in Resting Metabolic Rate (RMR)

It is important to consider the time of day difference in RMR when examining the diurnal rhythms in TEE, as RMR accounts for ~70% of humans’ TEE [30]. In this following section, we will describe studies that aim to examine the endogenous circadian rhythms and diurnal rhythms of RMR. We will also highlight studies that investigate the effects of circadian rhythm disruption and meal timing manipulation on RMR. As this is an emerging area, only a small number of studies to date have specifically examined the circadian rhythms of RMR. The endogenous circadian rhythm of physiological processes can only be inferred through the use of sophisticated metabolic studies, namely the constant routine protocol, and forced desynchrony protocol. 

Generally speaking, the constant routine protocol is regarded as one of best methods to measure circadian rhythms. In this protocol, external factors that influence circadian rhythms are maintained at a constant level, which is achieved by constant wakefulness, constant semi-recumbent posture, limited behavioural activity, dim light exposure and evenly spaced iso-caloric snacks [37]. Therefore, any rhythms measured are assumed to be a reflection of the pure circadian rhythm, generated by the endogenous circadian system [5]. Using a 34-h constant routine protocol, Krauchi et al. observed an endogenous circadian rhythm in RMR of male participants, with a nadir during the biological night (0000 h to 0600 h) and peak in the morning (0900 h and 1200 h) [38]. These findings are supported by Spengler et al. [39], who in a similar study design (41-h constant routine protocol in 10 men) also reported a circadian rhythm in metabolism. 

Although the constant routine protocol is typically the best method to infer circadian rhythms, the measurement of rhythmicity in RMR presents a unique situation in that the standard procedure of RMR measurement requires participants to have fasted for at least six hours prior [34]. This is never achieved in the constant routine protocol. As such, the patterns observed in these two studies [38,39] can only imply circadian rhythmicity in TEE (a combination of RMR and TEF). Considering this, the forced desynchrony protocol [40] may be a better method to elicit the circadian rhythm of RMR. Forced desynchrony studies change the day length so that it is sufficiently shorter or longer than the typical 24-h day to cause a desynchronisation between behavioural rhythms (i.e., sleep/wake and feed/fast) and the endogenous circadian rhythm. The study is then conducted over a series of these new “days,” allowing sleep and feeding behaviours to be measured at all phases of the circadian rhythm. This protocol allows mathematical separation of internal rhythms and behaviour while allowing participants to maintain a somewhat typical behavioural cycle (i.e., scheduled sleep and fasting periods) [5]. Using this protocol, Zitting et al. [41] reported a trough in RMR during the biological night (0100 h to 0500 h). This supports the findings from the constant routine studies, even though RMR measurement conditions used (rest and fast duration) were slightly different. Moreover, all studies reported a nadir in core body temperature at approximately 0500 h, further supporting low levels of metabolic thermogenesis during the biological night [38,41]. Some studies have tried to examine the time of day variation in RMR by conducting indirect calorimetry measurements at specific time points of the 24-h day. For example, diurnal studies that examined the effect of meal timing on TEF have reported on RMR at fasting (i.e., prior to administration of meal challenge) and found no difference between 0730 h and 1930 h [6,42]. It is important to note that a simple comparison between one daytime and one night time point is insufficient to describe the circadian rhythm of RMR. This is because in a typical diurnal study, the fast and sleep period prior to the night session differs to that in the morning. These study designs are outlined in Figure 2, but for a more comprehensive description please see a review by Poggiogalle et al. [5].

A small number of tightly controlled laboratory studies have examined the effects of acute circadian misalignment on both RMR and TEF, through simulated night shift work. These studies can help explain whether circadian misalignment, which is often experienced by shift workers, contributes to their increased risk of obesity [43]. Findings on RMR will be summarised in this section and findings on TEF will be discussed separately in a later section. In the study by Morris et al, no changes in RMR were observed after four simulated night shifts. However, RMR was only measured by indirect calorimetry at two time points of the day (0730 h and 1930 h) [42]. In contrast, in a study where TEE was measured for the entire study duration (four days) via whole-room indirect calorimetry, daily TEE was significantly reduced on the second and third simulated night shift, compared to day shift [4]. Given that minimal changes were observed for TEF, this finding is likely to indicate changes in RMR. If the reduced TEE was sustained and energy intake was not reduced, it could eventuate to positive energy balance and be a contributing factor towards the increased risk of obesity observed in shift workers [4]. The atypical schedules of shift work were closely simulated in a laboratory study conducted where participants were exposed to circadian disruption and sleep restriction concurrently for ~3 weeks [44]. In this study, circadian disruption was implemented by a forced desynchrony protocol, with a series of new “28-h days”. Participants were permitted to spend 6.5-h in bed each day, which was equivalent to a sleep restriction of 5.6-h per 24-h day, so we can be certain that an extended fast time was implemented as per standard RMR measurement guidelines. Note that the purpose of this forced desynchrony protocol was different to studies previously mentioned in this section, as mathematical modelling was not applied to separate the effects of the endogenous circadian rhythm. In this study an 8% reduction in RMR after 3 weeks of intervention was observed which the authors reported could translate into 5.6 kg of weight gain per year. However, due to the study design, this can only be said for individuals who experience both circadian disruption and sleep restriction concurrently, which may be the case for some shift workers.

Of particular clinical relevance are studies that examine the effects of changing mealtimes on RMR. Theoretically, it is more beneficial for weight maintenance/loss if RMR can be manipulated, rather than TEF, as RMR contributes to the largest proportion of TEE. In a cross-over study no difference in TEE between an early dinner (1900 h) and a late dinner (2230 h) intervention was observed [45]. However, this dietary change was only implemented for a single day, within a laboratory setting. In comparison, the RMR of women who underwent an early lunch (1300 h) and a late lunch (1630 h) intervention period were compared [46]. Each period was one week, and all meals were provided during the study and caloric intake for each individual was kept identical. A meal challenge (early or late lunch) was given at the end of each intervention period, depending on the intervention period just completed. The authors reported that delaying the timing of one meal (late lunch) was associated with a time-of-day variation in RMR with decreased RMR observed prior to the test meal challenge in late eaters. However, whilst lunch time was changed, dinner time was kept constant at 2000 h over the two intervention periods. Also, it is important to consider that there was a much higher energy load for the late eaters compared with the early eaters in the later part of the day (74% energy consumed after 1630 h compared with 27% energy), which was not discussed in the manuscript. 

The “Bath Breakfast Project” study compared the metabolic effects of breakfast eating to breakfast skipping (i.e., no food intake until after noon) for six weeks, in lean [47] and obese participants [48]. RMR was measured at 0800 h after an overnight fast (~10 h). No difference was found in RMR between the intervention groups in both cohorts of participants. However, as with most studies conducted in free-living conditions, confounders such as overall energy intake and other mealtimes could not be controlled [47,48]. In the lean cohort, overall daily energy intake was higher in the breakfast eating group compared to the breakfast skipping group. In a similar study, participants were provided with breakfast and morning tea items and participants were asked to consume all other meals at specific times [49]. Ten lean, female participants were involved in this cross-over study. During the two-week breakfast-eating period, participants consumed breakfast cereal between 0700 h and 0800 h, whereas in the two-week breakfast-omitting period, participants had their first meal of the day between 1030 h and 1100 h. As with the “Bath Breakfast Project”, RMR was measured in the morning (~0800 h) after an overnight fast and no difference was found in RMR between the two test periods [49].

Overall, there is some evidence to support that TEE follows a circadian rhythm, as observed in constant routine studies [38,39]. These studies suggest that the human body burns fewer calories during the biological night (~0000 h to 0600 h) [38,41]. Forced desynchrony studies suggest that a decrease in RMR may contribute to this lull in TEE during the night, however, this needs to be confirmed through additional forced desynchrony studies. Little is known about whether changes in meal timing can be used to manipulate RMR, as studies in current literature differ in the mealtime patterns tested. Studies in the current literature indicate that changes in daily meal timing are unable to manipulate RMR. However, variations in study protocol, such as mealtime patterns tested, health status of sample groups and duration of test periods, renders difficulty in drawing conclusions. Future studies should aim for longer intervention periods and keeping other mealtimes consistent during the study period. Moreover, comparing the difference in RMR between day workers and night shift workers (who habitually eat during the RMR nadir), may provide insights on the effects of long-term circadian misalignment on TEE.

## 5. Diurnal Variations in TEF

Multiple study designs have been employed aiming to elicit the circadian and diurnal variation in TEF, which attributes to approximately 10% of TEE. As discussed in the previous section, although a circadian rhythmicity of TEE can be observed through constant routine studies, it is impossible to differentiate whether this is a result of RMR and/or TEF. A forced desynchrony study [41] which examined circadian rhythms in RMR did not report on TEF, but reported on substrate oxidation, therefore its findings will be discussed in Section 6. In this section, diurnal variation in TEF will be discussed. Although these studies cannot differentiate the effects of the endogenous rhythm and external environmental/behavioural zeitgebers, they do provide an idea as to whether any time of day difference exists in TEF. Findings from a small number of studies which have examined the effects of circadian misalignment and meal timing manipulation will also be discussed.

The baseline circadian alignment protocol in the circadian alignment/misalignment study conducted by Morris et al. can be examined as a diurnal study [42]. The authors reported that TEF was 44% lower at 2000 h compared to 0800 h, following an identical meal. These findings were supported by authors of a similar study who reported a 380-kJ difference in TEF between night and day and concluded that this could have implications for long-term weight gain, if a pattern of night-time eating was sustained over time [6]. It is important to note that as per typical diurnal studies, it is difficult to control for effects of behavioural cycles. For example, in the study by Morris et al., a 12-h fast preceded the morning session, while the night session was preceded by an 8.5-h fast. Moreover, participants had an overnight sleep occasion prior to the morning session, which was absent prior to the night session [42]. These effects were minimised by Bo et al. by asking participants to consume a standardised meal 8-h prior to testing, followed by a 6-h rest in bed. However, this meant that participants had an energy dense (4.9-MJ) standardised meal at 0000 h prior to the morning session (0800 h), a time when they may not normally be awake and eating [6].

In a study that compared TEF after provision of meals in the morning compared to the afternoon no difference in TEF was observed between the morning and afternoon [50]. Morning and afternoon measurements of RMR and TEF were undertaken on separate days to exclude any residual effect from the morning assessment. Measurements were also run repeatedly (three morning and three afternoon sessions) to minimise obscuring any diurnal variation that may be present. Whilst conditions for TEF were kept constant between morning and afternoon (provided the same test meal, constant room temperature, and supine), the post-absorptive period differed between morning and afternoon (12–14 h on the morning tests vs. 6–7 h in the afternoon). A similar study in 11 obese boys reported no significant differences in TEF following identical meals consumed at 0730 h and 1200 h [51]. Given that both of these studies examined morning versus afternoon TEF, it is plausible to suggest that variations in TEF are less pronounced between these two daytime periods (i.e., 4–5 h apart) and may require much larger sample sizes to detect differences when compared with the more extreme comparisons of morning versus night (i.e., 10–12 h apart) periods.

A small number of laboratory studies have examined the effects of acute circadian misalignment on TEF, through simulated night shift work. In a 3-day simulated night shift study with 14 participants, McHill et al. reported that TEF post-dinner on the 1st day of night shift (2230 h) was significantly lower than that during baseline (“day shift” at 1830 h). More importantly, the daily TEE on the 2nd and 3rd night shift was reduced compared to “day shift”. The authors concluded that this difference of ~210 kJ/day can lead to weight gain over time in populations such as shift workers, who habitually eat during the night. [4]. In contrast, in a study of 11 participants (3-day simulated night shift), no effect of circadian misalignment of TEF was reported [42]. In a separate study in actual shift workers, in which participants consumed a snack which made up 20% of total energy intake at 0100 h, 0900 h and 1700 h, the snack consumed at 0900 h was found to produce a significantly higher TEF than the snack consumed at 1700 h or 0100 h [52]. Although these findings are supportive of those reported by McHill et al., it is important to note that this study had a small sample size (nine participants) who were potentially circadian-disrupted given their role as shift workers, meaning their results may not necessarily be translatable to the wider, non-shift working population. 

Of particular clinical interest are studies that report on the impact of changing mealtimes on TEF. In a study that compared TEE over a 24-h period whereby participants were either fed every four hours (six meals in total) or three larger meals followed by an overnight fast a difference in TEF was observed [53]. Whilst it was not surprising that EE overnight was higher in the study arm where participants ate (in comparison to fasting), TEE over the 24-h period was no different. The authors conclude that there appears to be no advantage to consuming three larger meals per day compared to smaller meals spread across a 24-h period. However, it could also be concluded that neither strategy has an additional benefit based on assessment of TEE only. Moreover, having six evenly spaced meals in the 24-h period is not typical in both day work and shift work populations. In a study described earlier, researchers examined whether changes in mealtime impacted TEE over a 24-h period. No difference in the TEF after a “normal” (1900 h) or “late” (2230 h) evening meal was observed [45]. However, both meals in this study were provided during the “evening time”, which is potentially a time already associated with reduced TEE. Further, this change in mealtime was only implemented for a single day. In a crossover study, female participants were provided with early lunch (1300 h) and late lunch (1630 h), each for a one-week period [46]. The “late” lunch protocol did not have an effect on TEF, however, a lower RMR (prior to test meal administration) was observed.

The collective findings presented above (lowered TEF at night, reduced TEE at night and reduced RMR post night shift) demonstrate that circadian disruption, as frequently occurs in shift workers may cause moderate but potentially noteworthy adaptive reductions in TEE. This provides a partial explanation for the reported increased weight gain observed in shift working populations. 

## 6. Changes in Substrate Oxidation and Fuel Storage Across the Day

The role of substrate source in determining the magnitude of the TEF is an important consideration to explain the effect of time of day on the rate of utilisation versus storage of dietary components including macronutrients. 

A number of studies have examined circadian (or diurnal) rhythms in substrate oxidation after administration of iso-energetic and iso-macronutrient snacks or meals. A clear circadian variation was reported in rates of protein oxidation; urea concentration (measured over a 30-h period in seven men undergoing a constant routine protocol) was lowest at 2300–0200 h rising to peak at 0800–1100 h [38]. The same study yielded no circadian variations in carbohydrate and fat oxidation rates across the 24-h period. More recent studies have found evidence for differential rates of substrate oxidation across the day. In a forced desynchony study, oxidation of both carbohydrates and fat oscillated over the participant’s circadian phase, but displayed different peaks and nadirs [41]. It was found that carbohydrate oxidation was highest during the biological morning and lowest during the biological evening. Conversely, lipid oxidation peaked during the biological evening with the nadir occurring during the biological morning. Similarly, using a circadian misalignment protocol, researchers observed that carbohydrate oxidation was 10% lower in the biological evening compared to morning, however no significant circadian rhythms in lipid oxidation were detected [54]. Whilst a separate study reported a significant decrease in after-meal carbohydrate oxidation and increase in after-meal fat oxidation at night compared to the morning in a cross-over study of 20 healthy participants [6]. This study was not set up to measure a circadian response, rather is only indicative of a diurnal response in substrate oxidation. 

Two studies have been conducted that have compared the metabolic response to meals of differing macronutrient composition. In one study differences in the TEF between two 36-h dietary patterns were examined in an in-laboratory protocol utilising a respiration chamber [55]. The use of a respiration chamber enables participants to spend the night in the chamber and be free living under test conditions (i.e., not restricted by a ventilated hood). The authors reported that the total TEF was greater after a high protein diet (29% energy from protein, 10% fat, 61% carbohydrate), compared to an isocaloric, high fat diet (9% energy from protein, 61% fat, 30% carbohydrate). However, TEF was not measured post-meal in this study, instead, it was derived from subtracting sleeping metabolic rate from the RMR. Therefore, it is difficult to determine whether macronutrient differences of the two diets affected TEF, based on the TEF definition used in this review. Further, there was no significant difference found in TEE between the two diets. In a second study, using an experimental cross-over design, researchers examined differences in metabolic response to either a high fat or high carbohydrate diet, provided as six isocaloric meals over a 24-h period where participants were kept awake for the 24-h period [56]. Overall, TEE was greater after the high fat diet compared with the high carbohydrate diet. No difference in EE was found between day and night, nor was there evidence to suggest the macronutrient composition of the diets affected the diurnal pattern of TEE. A diurnal pattern in fat oxidation was observed only in the high fat diet (fat oxidation was reduced at 0800 h); significant diurnal patterns were not found for carbohydrate or protein oxidation for either meals. A recent cross-over study examining the effect of meal timing on TEE, over a 24-h period, observed no difference in energy expenditure when participants consumed all their food within a 6-h or a 12-h period [57]. Energy expenditure was measured using indirect calorimetry following standard conditions. The authors concluded that whilst there was no effect of timing of food intake on TEE, eating within a shortened window of time (6-h vs. 12-h) did increase TEF, supportive of a diurnal rhythm for TEF. 

Overall, these findings suggest that macronutrient composition of evening meals may play a role in the efficiency of substrate utilisation during the biological night and the preliminary evidence suggests favouring meals higher in fat over carbohydrates. However, whether these differences in substrate oxidation rates will affect TEE remains unclear. Further, the quality of the fat consumed should be considered as some studies have suggested that the inclusion of unsaturated fats compared with saturated fat have the potential to increase fat oxidation [58,59]. The proposed mechanisms in these studies involved upregulated expression of uncoupling proteins (UCP’s) in skeletal muscle, namely UCP-2 [60,61] and UCP-3 [62], as well as peroxisomal Acyl-CoA [60,62] in skeletal, liver and cardiac muscle and elevated PPAR-γ and PPAR-α [63,64]. Increased expression of UCPs facilitates the release of energy via thermogenesis; increasing the expression of Acyl-CoA promotes fat oxidation via the peroxisomal pathway; and elevated PPAR expression upregulates mitochondrial carnitine palmitoyl transferase 1 (CPT-1) [65] which can in turn promote β-oxidation. It is important to note, however, that these mechanisms have not been investigated specifically at night.

Lifestyle stressors such as altered sleep and eating patterns that may disturb the host (i.e., human) circadian system also adversely influence the gut microbiome. The composition of the gut microbiota also follows a diurnal oscillating pattern [66] and partly regulates the extent to which energy is derived from foods [67,68].

If gut microbiota are depleted and there is disruption to the peripheral circadian clock within the intestinal epithelial cells [69], this may result in reduced fuel utilisation. Circadian misalignment due to transmeridian travel/jetlag and late or night-time eating negatively impact the abundance and species diversity of the human gut and salivary microbiota, potentially predisposing individuals to increased risk of weight gain and exacerbation of glucose intolerance [66,70]. Short chain fatty acids (SCFAs) (primarily acetate, butyrate and propionate) are byproducts of microbial fermentation in the colon, which have important functions both within the intestinal lumen and in peripheral tissues. In murine studies, butyrate induces lipolysis, fatty acid oxidation and thermogenesis, while acetate enhances beiging in white adipose tissue (WAT), which in turn, could enhance TEE [71,72]. In humans, however, the relationship between SCFAs and browning of WAT is inconsistent. Furthermore, the combination of gut bacteria can impact energy storage. Studies in mice indicate peaks in the abundance of the bacterial phylum, *Firmicutes*, immediately after meals, with the more beneficial phyla *Bacteroidetes* and *Proteobacteria* only able to bloom during periods of fasting [73]. A 20% increase in *Firmicutes* with a corresponding decrease in *Bacteroides* was associated with an increased energy harvest from the host diet of 630 kJ per day, potentially resulting in a body weight gain of 5 kg over one year [68]. We can speculate that consequent disruptions to microbiota-mediated functions, as a result of circadian disruption, such as decreased production of butyrate, may in turn impact substrate oxidation and therefore energy regulation in the host. Further studies are needed to confirm this using controlled circadian protocols comparing the effects of isocaloric meals. 

Whilst diurnal changes to macronutrient storage have infrequently been considered within the context of TEE and weight change, a temporal pattern to adipose tissue accumulation over a standard day (i.e., three meals at usual clock times) has been observed [74]. This study suggests an increased efficiency to store fat later in the day in men (*n* = 8) following a standard mealtime pattern. Whether this same temporal pattern in fat storage occurs when people invert their mealtimes to occur during the night such as in shift work is not yet known. It is plausible that regulation of fat storage is influenced by meal timing, however, a clear pattern has not yet emerged to identify which combination of factors is more likely to promote fat storage. It is also worth noting whether these findings are indicative of a second meal effect, as after the second meal there was less suppression of NEFAs and a tendency for a smaller insulin AUC. A study which included people fasting during daylight hours for the religious celebration of Ramadan [75] reported a reduced body weight in those fasting during the day compared with a matched control group who fasted overnight, however this may in part be due to a small reduction in overall energy intake. In this same study, in participants who fasted during the day, fat oxidation was measured using indirect calorimetry post-prandial in the morning (prior to commencing the daily fast) and in the evening and found that fat oxidation was lower at night, however there was no detail on meal composition which confounds the interpretation of these data. In a study of 11 healthy women it was reported that nighttime snacking reduces whole body fat oxidation [76]. The snack was consumed either at 1000 h or 2300 h for 13 days and a reduced fat oxidation was reported following the snack consumption at night compared to the same snack consumed in the morning. However, a limitation of this study was that food consumption outside of the specified snacks was not standardised.

There is evidence to suggest that the balance between storage and utilisation changes with transitions from day shift to night shift, but this shifts again with repeated consecutive night shifts [4]. In a study of men and women (*n* = 14) that compared to baseline (a day time measure prior to simulated night shift), fat utilisation increased on the first two nights of simulated night shift (by 8% and 18% respectively), which then returned to baseline levels on the third night shift. The authors suggested this may, in part, be due to prolonged wakefulness on transition days leading to an overall greater TEE. Moreover, participants were unable to adjust their energy intake due to the controlled experimental conditions which may have created an artificial negative energy balance during these prolonged periods of wakefulness. Alternatively, this could also suggest that the timing of meal intake over the three nights has altered peripheral circadian regulation of metabolism. Extensive investigation is required to tease out the mechanisms for increased weight gain linked with later day eating and shift work. Whist some evidence suggests that reduced TEE at night may explain the propensity to gain weight, the mechanisms driving this are not clear. Moreover, there is a need to better understand what main factors promote fat storage over the 24-h cycle.

## 7. Are Observational Studies Suggestive of an Effect of Meal Timing on Body Weight?

Reported variations in RMR and TEF across the day may help with the interpretation of observational studies that have explored the association between meal timing and weight status. Even after adjustment for total energy intake, eating a greater proportion of daily energy at certain times of the day is associated with increased risk of weight gain or obesity and overweight. The following sections describe data from observational and experimental studies that imply an effect of meal timing on weight.

In a cohort recruited in 2008–2010 and followed up 3.5 years later, consuming a greater proportion of energy at lunch (data divided into quartiles of % energy intake) appeared to reduce the risk of weight gain at follow-up (Odds Ratio (OR) = 0.62, 95% Confidence Intervals (CI): 0.47; 0.80) in 4243 individuals [77]. However, follow up weight was self-reported, and no association was found between the percentage of energy intake at the rest of the eating occasions (i.e., breakfast and/or dinner) and weight gain. In a separate cohort study, participants who consumed ≥48% of their daily energy intake at dinner (data divided into tertiles of % energy intake) were twice as likely to be obese at 6-year follow up, even after adjustment for variations in total energy intake, physical activity and BMI at baseline (OR =2.33; 95% CI: 1.17; 4.65) [78]. The authors suggest that increased TEF in the morning compared to the evening could be one possible mechanism behind this association, but this was not measured. 

In a separate study of 239 participants, those who consumed ≥33% of total energy in the evening (compared to those who consumed <33%) were twice as likely to be overweight or obese (OR = 2.00; 95% CI: 1.03; 3.89) [79]. These findings were also adjusted for variations in total energy intake and physical activity. While the results of this study are supportive of the aforementioned studies, the cross-sectional nature of these results makes it difficult to draw a clear link between meal timing and weight status. Further, the association between overweight/obesity and the higher evening energy consumption was non-significant amongst who the authors deemed ‘true reporters’ (participants who accurately reported ±25% of their TEE, as measured by doubly labelled water) (OR = 2.10; 95% CI: 0.60; 7.29). The authors proposed that individuals who consume a greater energy load in the daytime (as opposed to evening) have a feeling of satiation which carries over to the evening meal, which subsequently reduces energy intake at night [79]. Meanwhile, individuals who consume a larger energy load in the evening are unlikely to carry over a feeling of satiation to the next day, and consequently consume a greater amount of energy over time. The authors did not suggest time-of-day variations in TEF as a possible mechanism behind their findings nor did they measure satiety. Furthermore, the authors have not commented on the greater fasting time experienced by those eating late into the night as they fast overnight before their next meal. 

A clear limitation of a number of these studies is that subjects’ patterns of energy distribution are based on a single food diary or dietary history [77,78,79]. Thus, these results are based on the assumption that participants did not change their eating habits during extended follow-up periods which should be confirmed in future studies. Furthermore, the distribution of macronutrient intake in the diet is not considered and how this may impact TEE [80].

## 8. Using Changes in Meal Timing to Enhance Weight Loss

Identified weight loss interventions that have considered time of day (meal timing) in their reporting lend some support to the observational data reported above and are described below. These weight loss interventions have reported that consuming a higher proportion of energy in the morning produces better weight loss outcomes, however these studies did not specifically measure EE. 

No influence from the timing of breakfast or dinner on weight loss success of participants was observed in a weight loss study, however, an association with the timing of lunch was observed [81]. Those classed as early eaters (prior to 1500 h) showed greater weight loss success than those classed as late eaters (after 1500 h). Although subjects recorded their dietary intakes for the whole intervention, one randomly selected week of dietary data was used to cover the 20 weeks of intervention and used for data analysis. The rationale for selecting 1500 h as a cut-off for lunch was not clear. Although this study was in a Mediterranean population and late lunch may be typical, these findings may not be generalisable to other populations where eating up to 1500 h might be considered to be a late lunch. That being said, similar results are reported in a population of post bariatric surgery patients [82]. No difference was reported in total energy intake between participants post-surgery, however, those who responded the best with respect to weight loss reported consuming their main meal (lunch) prior to 1500 h. In a study of 85 women involved in a weight loss intervention, the authors found that the best responders had lower day time body temperatures [83]. Body temperature (measured at the wrist) has been found to be a practical and effective proxy measure of circadian rhythms [83]. This could indicate that meal timing and weight loss success may be influenced by individual variations in circadian rhythms and generalised advice regarding meal timing during weight loss may not be appropriate.

Intervention trials to promote weight loss lasting 12 and 8 weeks respectively, showed that when the majority of energy (50%) was consumed during the breakfast meal, participants lost more weight compared to those who consumed the majority of energy (50%) at dinner [84,85]. Both ‘breakfast’ and ‘dinner’ groups in these studies were provided isocaloric, weight loss diets. Jakubowicz et al., who conducted their study in a free-living population, found 2.5 times the weight loss in a ‘breakfast’ group (−8.7 ± 1.4 kg) compared to ‘dinner’ group (−3.6 ± 1.5 kg, *p* < 0.0001) [84]. These findings, consistent with the aforementioned observational studies, suggest that energy loading earlier in the day is optimal for weight control.

However, the impact of energy loading at nighttime on weight outcomes is inconsistent. In a study that investigated the effects of consuming 70% of daily energy in two morning meals compared to two evening meals [86], larger morning meals were associated with slightly better weight loss outcomes than larger evening meals (−3.9 ± 0.19 vs. −3.27 ± 0.26 kg, *p* < 0.01). However, larger evening meals resulted in better maintenance of fat-free mass than larger morning meals (−0.25 ± 0.16 vs. −1.28 ± 0.14 kg, *p* < 0.001). The authors hypothesise that the attenuation of fat-free mass loss may have been due to higher rates of muscle glycogen synthesis overnight as a result of higher carbohydrate consumption in the evening. According to previous studies by Blom et al. and Clarys et al., this is indeed a plausible explanation for the 25% difference in fat free mass observed between the group consuming larger morning meals and the group consuming larger evening meals [87,88]. In a separate weight loss study, two hypocaloric diets with different energy loading were tested in men with obesity [89]. One involved consuming 50%, 35% and 15% of daily energy at breakfast, lunch and dinner respectively. The other involved consuming 15%, 35% and 50% of daily energy at breakfast, lunch and dinner, respectively. Although participants lost weight in both groups, there were no significant differences in the percentage or absolute amount of weight lost between those who had consumed more energy at breakfast versus dinner. Of note, however, this study was powered to detect differences in serotonin and dopamine binding as a result of energy loading at breakfast versus dinner during a weight-loss intervention. The study was not specifically powered to detect the differences in weight loss between the breakfast versus dinner groups.

Overall, the small number of studies that have explored the relationship between weight status and meal timing suggest that consuming the majority of energy earlier in the day may promote more successful weight loss or long-term weight control, but these findings are inconclusive. Circadian variation in TEE, primarily as TEF, was touted as a possible cause, however, as EE was not measured in the majority of studies the mechanism was speculative. 

## 9. Sleep and TEE Across the Day 

Whilst the focus of this review has been to investigate the literature exploring changes in TEE across the 24-h day with the focus on changes to RMR and TEF, another factor that may also directly or indirectly impact TEE is sleep restriction. 

Chronically restricted sleep is a risk factor for weight gain and obesity [90,91]. A focus for research has been investigating the mechanism(s) for this relationship, specifically interrogating whether sleep loss influences energy balance via changes in intake, expenditure, or both. Findings in human studies show analogous changes in TEE and intake, however, the resulting energy balance is in favour of weight gain.

A long-time hypothesised function of sleep is to conserve energy, and this is supported by research measuring EE during sleep [92]. While some studies of sleep loss have found increases in daily TEE during sleep restriction [93] and total sleep deprivation [94], others have found mixed results [94,95]. It has therefore been argued that while sleep loss may result in increased TEE in humans, the increases seen in humans are at lower levels than those observed in rodent models [96]. 

Sleep restriction in humans may result in increased energy intake through increased energy required to sustain the increases in expenditure, as well as increased time available to eat [97]. Studies also suggest that changes in hormones that influence appetite and satiety may play a part; leptin (anorexigenic) and ghrelin (orexigenic) [98,99]. However, studies have not shown consistent effects of sleep loss on these hormones [94,100,101,102]. More consistent results have been found in the relationship between sleep loss and an increased consumption of energy-dense foods in both shift workers [103,104,105] and non-shift workers [100,106,107,108,109]. 

Overall, research to date indicates that in humans, sleep restriction results in increases in energy intake that outweigh increases in expenditure, resulting in positive energy balance [93,94].

## 10. Conclusions

In general, the papers reviewed suggest that prioritising energy intake earlier in the day may support increased energy expenditure [81,110] and that morning-loaded energy distribution is a feasible strategy for weight control in people who are in circadian alignment. The studies that promote this view, whilst being well conducted, focus primarily on TEF and vary considerably in their methodological approaches, including time of meal consumption, fasting periods and caloric load of test meals. Whilst these variables need to be taken into account in order to fully understand the impact of daily rhythms on TEF, the lack of homogenous study designs to date precludes firmer conclusions being made. Currently, only a cursory consideration has been given to diurnal and/or circadian changes in RMR across the day and as such conclusions cannot currently be drawn as to the extent that meal timing impacts RMR. Other contributing factors such as hormone secretion, sleep and gut microbiota are also important to consider, but to date also lack attention in the literature. This review is the first to consider all these contributing factors together in one synthesised review. 

The actual proportion of additional energy expended during the day compared with night time from TEF studies would not provide a substantial weight loss and caution should be taken in drawing these conclusions. Incorporation of an assessment of RMR into such studies may provide additional evidence. It is feasible, however, that an ‘intentional approach to eating’, as described by the American Heart Association, which focuses on meal timing and frequency could improve risk factor management [111]. Avoiding eating at night, where possible, could impact on overall energy intake and also avoid the metabolic perturbations observed as a result of night time eating. Furthermore, daytime-loaded energy intake appears to be more effectively metabolised, with TEE being less efficient during the later hours. 

In order to effectively assess the contribution of the endogenous circadian system on energy balance and subsequent metabolic health, it is important to undertake studies that target meal timing and frequency in conjunction with a more thorough assessment of energy balance. Ensuring the appropriate laboratory protocol is utilised to determine whether a response is indeed circadian or diurnal will add to the challenge and costs of such studies. For now, dietary guidance for both timing and quality of foods consumed is a priority for those that have no choice but to eat at night.
